# In Vitro and Clinical Compassionate Use Experiences with the Drug-Repurposing Approach CUSP9v3 in Glioblastoma

**DOI:** 10.3390/ph14121241

**Published:** 2021-11-29

**Authors:** Marc-Eric Halatsch, Annika Dwucet, Carl Julius Schmidt, Julius Mühlnickel, Tim Heiland, Katharina Zeiler, Markus D. Siegelin, Richard Eric Kast, Georg Karpel-Massler

**Affiliations:** 1 Department of Neurological Surgery, Ulm University Medical Center, 89081 Ulm, Germany; annika.dwucet@uniklinik-ulm.de (A.D.); carl.schmidt@uni-ulm.de (C.J.S.); julius.muehlnickel@med.uni-duesseldorf.de (J.M.); tim.heiland@uni-ulm.de (T.H.); katharina.zeiler@uni-ulm.de (K.Z.); 2 Department of Neurological Surgery, Cantonal Hospital of Winterthur, 8401 Winterthur, Switzerland; 3 Department of Pathology and Cell Biology, Columbia University Irving Medical Center, New York, NY 10032, USA; ms4169@cumc.columbia.edu; 4 IIAIGC Study Center, Burlington, VT 05408, USA; richarderickast@gmail.com

**Keywords:** CUSP9*, CUSP9v3, glioblastoma, drug repurposing, compassionate use

## Abstract

Background: Glioblastoma represents the most common primary brain tumor in adults. Despite technological advances, patients with this disease typically die within 1–2 years after diagnosis. In the search for novel therapeutics, drug repurposing has emerged as an alternative to traditional drug development pipelines, potentially facilitating and expediting the transition from drug discovery to clinical application. In a drug repurposing effort, the original CUSP9 and its derivatives CUSP9* and CUSP9v3 were developed as combinations of nine non-oncological drugs combined with metronomic low-dose temozolomide. Methods: In this work, we performed pre-clinical testing of CUSP9v3 in different established, primary cultured and stem-like glioblastoma models. In addition, eight patients with heavily pre-treated recurrent glioblastoma received the CUSP9v3 regime on a compassionate use basis in a last-ditch effort. Results: CUSP9v3 had profound antiproliferative and pro-apoptotic effects across all tested glioblastoma models. Moreover, the cells’ migratory capacity and ability to form tumor spheres was drastically reduced. In vitro, additional treatment with temozolomide did not significantly enhance the antineoplastic activity of CUSP9v3. CUSP9v3 was well-tolerated with the most frequent grade 3 or 4 adverse events being increased hepatic enzyme levels. Conclusions: CUSP9v3 displays a strong anti-proliferative and anti-migratory activity in vitro and seems to be safe to apply to patients. These data have prompted further investigation of CUSP9v3 in a phase Ib/IIa clinical trial (NCT02770378).

## 1. Introduction

Glioblastoma is one of the most common brain tumors, presumably derived from glial cells [1]. The malignant nature of this disease entails a fierce clinical course despite first-line therapy that includes neurologically safe maximal resection, radiochemotherapy and tumor-treating fields [2,3]. In over 500 clinical trials of various old and new cytotoxic drugs in patients with glioblastoma over the last ten years, little progress in lengthening overall survival has been reported. Intratumoral heterogeneity as a result of early clonal diversion constitutes a key factor of therapeutic resistance with the dominant clone at diagnosis generally not being a linear ancestor of the dominant clone at recurrence [4,5]. The specific nature of such clonal diversion and the signaling pathways involved is currently not predictable. Along these lines, a single-drug/single-target strategy does not seem to constitute a rational strategy to overcome the core problem. We therefore adopted a broader concept of signaling inhibition with the CUSP9 approach [6,7].

Drug repurposing has gained significant attention as an alternative to the traditional drug development process [8,9,10]. This concept relies on identifying drugs that are already approved and marketed for other indications but that also possess anti-glioblastoma activity due to interference with dysregulated signaling pathways that are physiologically involved in cell growth, cell death or cell migration. Because these drugs are in clinical use, their safety profiles are well-characterized, potentially allowing to accelerate their clinical application for a different medical condition at reduced financial cost.

CUSP9 is a therapeutic strategy merging a polypharmaceutical, multitargeting approach with the concept of drug repurposing in order to address the intratumoral heterogeneity of glioblastoma and to potentially allow for fast clinical translation [6,7]. Based on rational criteria, nine already-marketed non-oncological drugs were identified which share as common features (1) a sound pharmacological characterization, (2) inhibitory effects on growth-promoting signaling pathways and (3) a low probability of adding to the side effect burden that is already present (Table 1 provides an overview of the different CUSP9 regimes).

The resulting original CUSP9 drug cocktail included the anti-emetic aprepitant, the antimalarial artesunate, the antirheumatic auranofin, the antihypertensive captopril, the alcohol consumption deterrent disulfiram, the food supplement copper gluconate, the antifungal ketoconazole, the antiretroviral nelfinavir and the antidepressant sertraline, all combined with low-dose temozolomide, the current mainstay cytotoxic drug during first-line treatment of glioblastoma. Due to the fact that ketoconazole was delisted by the European Medicines Agency, itraconazole was chosen as a replacement. When the production of nelfinavir was stopped, it was replaced by ritonavir. In addition, copper gluconate was no longer considered essential for disulfiram activity in vivo and the non-steroidal anti-inflammatory drug (NSAID) celecoxib was introduced instead. These modifications resulted in the treatment regimen CUSP9* [7]. A further refinement, replacing artesunate by the antibiotic minocycline due to concerns of enhanced artesunate toxicity in the combination setting, led to the development of CUSP9v3.

In this study, we present our preparatory in vitro work testing the antiglioblastoma activity of CUSP9v3 in different glioblastoma model systems. We also report on the preliminary safety of compassionate use CUSP9v3 offered to eight heavily pre-treated patients with recurrent glioblastoma.

## 2. Results

### 2.1. CUSP9v3 Reduces the Viability of Glioblastoma Cells

**Figure 1:**
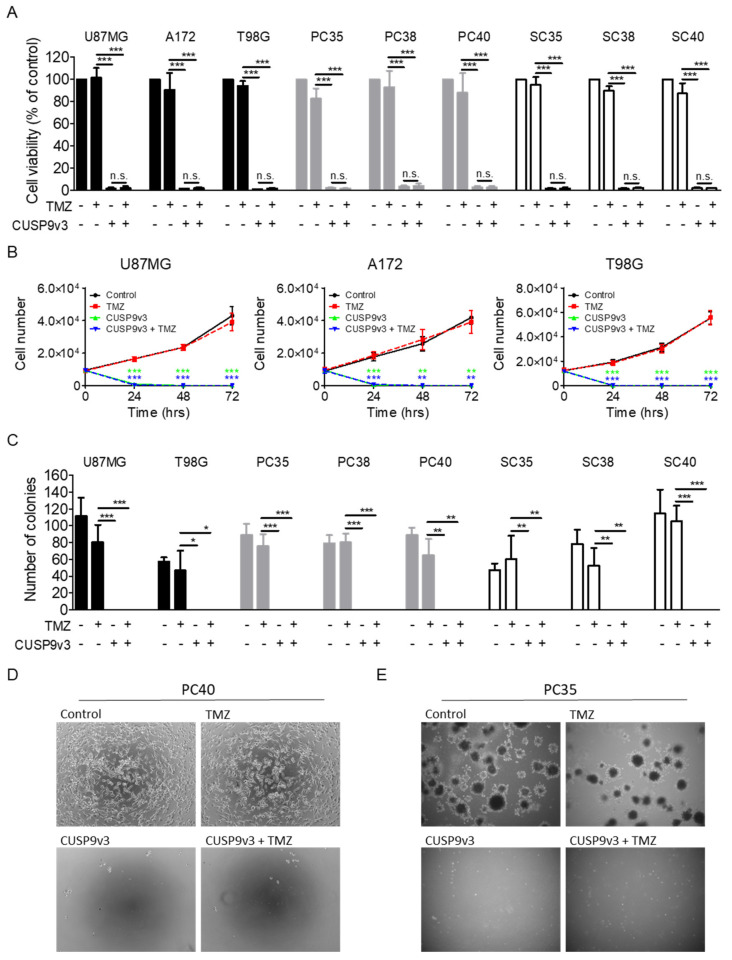
(**A**–**C**), Data represent three independent experiments and are presented as mean and standard deviation. * *p* < 0.05, ** *p* < 0.01, *** *p* < 0.005 versus TMZ. (**A**), U87MG, A172, T98G established glioblastoma cell lines, PC35, PC38 and PC40 glioblastoma primary cultures and SC35, SC38 and SC40 glioblastoma stem-like cells were treated with solvent, temozolomide (TMZ), CUSP9v3 or CUSP9v3 plus TMZ for 144 h. Cellular viability was determined by MTT assays. (**B**), U87MG, A172 and T98G cells were treated for 72 h as indicated. Cell numbers were determined at indicated time points. (**C**), The indicated glioblastoma cells were seeded in soft agar and treated either with solvent, TMZ, CUSP9v3 or CUSP9v3 plus TMZ for 21 d. Inhibitory effects on anchorage-independent growth were assessed by counting colonies that formed in soft agar. (**D**), Representative microphotographs of PC40 glioblastoma cells treated for 144 h as indicated. Magnification 10×. (**E**), Representative microphotographs of PC35 colonies in soft agar treated for 21 d as indicated. Magnification 4×.

We hypothesized that based on existing evidence, the non-oncological drugs included in the CUSP9v3 regimen would negatively affect the proliferation of glioblastoma cells at plasma concentrations achievable in humans. To test this hypothesis, we treated different established glioblastoma cell lines, primary cultures and glioblastoma stem-like cells with CUSP9v3 prior to performing MTT assays. While treatment with temozolomide alone for 144 h induced only a slight decrease of the cellular viability in all glioblastoma models tested, treatment with CUSP9v3 without temozolomide resulted in a complete or nearly complete suppression of tumor cell viability (Figure 1A,D). Addition of temozolomide did not further enhance the antiproliferative activity of CUSP9v3. In line with this finding, CUSP9v3 exposure resulted in marked reduction of tumor cell numbers as early as 24 h after treatment (Figure 1B,D). Again, additional treatment with temozolomide did not increase the activity of CUSP9v3 alone.

We further examined how a decrease of the CUSP9v3 drug concentrations to 1/10 of the original doses (CUSP9v3 1/10) affects the proliferation of glioblastoma cells (Figure S1A,B). While U87MG, PC35, PC38 and PC40 still showed a strong response towards the dose-reduced CUSP9v3 regimen, it had only little effect on the glioblastoma stem-like cells SC35, SC38 and SC40 as well as on A172 and T98G cells. Additional treatment with temozolomide did not significantly enhance the antiproliferative activity of CUSP9v3 1/10.

### 2.2. CUSP9v3 Abolishes Anchorage-Independent Growth of Glioblastoma Cells

The ability to form colonies in soft agar is a typical feature of many cancer cells. We therefore examined whether treatment with CUSP9v3 would affect this ability in glioblastoma cells. As shown in Figure 1C,E, treatment with the standard glioblastoma chemotherapeutic agent temozolomide alone did not affect colony formation under the conditions we used. In contrast, treatment with CUSP9v3 completely inhibited anchorage-independent growth—an effect which could not be further enhanced by additional treatment with temozolomide. Again, this effect was observed across established glioblastoma cell lines, primary cultures and glioblastoma stem-like cells.

### 2.3. CUSP9v3 Inhibits 3-Dimensional Tumor Growth

**Figure 2:**
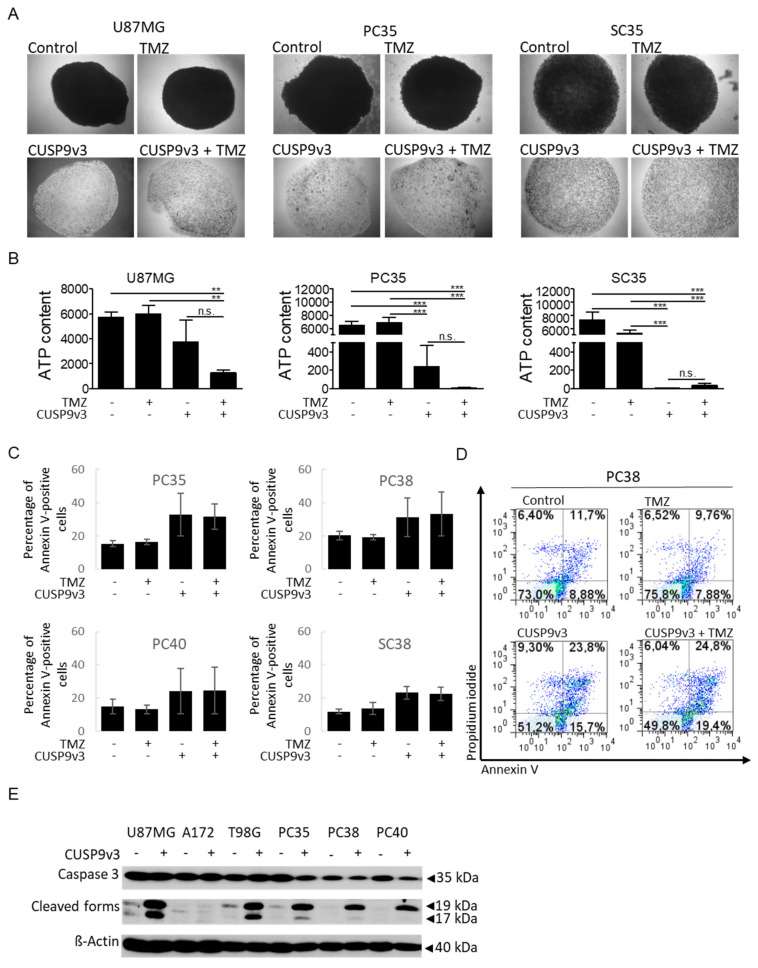
(**A**), Representative microphotographs of U87MG, PC35 and SC35 spheroids treated 1 h after seeding followed by treatments on d7, d9 and d12. 4× magnification. (**B**), Spheroids of U87MG, PC35 and SC35 glioblastoma cells were grown for 7 d prior to treatments on d7, d9 and d12. CellTiter-Glo^®^ assays were performed to determine the ATP content of the cells comprising the spheroids. Data are representative for two independent experiments. Columns: mean. Bars: standard deviation. ** *p* < 0.01, *** *p* < 0.005. (**C**), PC35, PC38, PC40 and SC 38 cells were treated for 6 h as indicated. Annexin V/PI staining was performed to determine the fraction of Annexin V-positive cells. Data represent three independent experiments. Columns: mean. Bars: standard deviation. (**D**), Representative flow plots of PC38 cells treated for 6 h as indicated prior to staining for Annexin V/Propidium iodide (PI) and flowcytometric analysis. (**E**), U87MG, A172, T98G, PC35, PC38 and PC40 glioblastoma cells were treated with solvent or CUSP9v3 for 6 h. Whole cell extracts were collected and Western blot analysis was performed for Caspase 3 and its cleaved forms. β-Actin served as loading control.

As tumors in patients grow spatially in three dimensions, we examined whether the CUSP9v3 regimen would be able to impair tumor cell proliferation in a more complex 3-dimensional model, i.e., spheroid assays. First, we examined whether treatment with CUSP9v3 is able to prevent the formation of spheroids. In this chemoprevention model, treatment was started already one hour after seeding the spheroids. As shown in Figure 2A, treatment with CUSP9v3 in the presence or absence of temozolomide resulted in significant impairment of spheroid formation. Second, we assessed whether CUSP9v3 would also be able to impair the growth of already established spheroids (Figure 2B). For this purpose, spheroids were allowed to grow for 7 days before starting the treatments. In spheroids derived from PC35 and SC35 cells, treatment with CUSP9v3 led to a marked decrease in spheroid growth as witnessed by a significant reduction in ATP content. In spheroids derived from U87MG, decreases in ATP content were also noted, however, not in a statistically significant manner. Additional treatment with temozolomide led to further enhanced reductions of ATP contents in spheroids from U87MG and PC35 cells when compared to treatment with CUSP9v3 alone (Figure 2B), although the observed differences were statistically not significant.

### 2.4. CUSP9v3 Leads to Enhanced Apoptosis

Microscopic analyses revealed that human glioblastoma cells treated with CUSP9v3 displayed typical morphologic features of apoptosis such as a rounding of cells and the formation of blebs. In addition, our cell count analyses revealed a significant reduction of cell numbers already 24 h after treatment with CUSP9v3. We therefore used Annexin V/Propidium iodide assays to assess whether CUSP9v3 leads to an increase of apoptotic cell death. As shown in Figure 2C,D, treatment with temozolomide alone did not increase the fraction of Annexin V-positive cells when compared to control. In contrast, treatment with CUSP9v3 led to a marked increase of the fraction of Annexin V-positive cells as early as 6 h after treatment. Additional treatment with temozolomide did not further enhance this effect. In accordance with this finding, treatment with CUSP9v3 led to significantly enhanced cleavage of effector caspase 3 in established and primary cultured glioblastoma cells (Figure 2E).

### 2.5. CUSP9v3 Impairs Non-Directed and Directed Movement of Glioblastoma Cells

**Figure 3:**
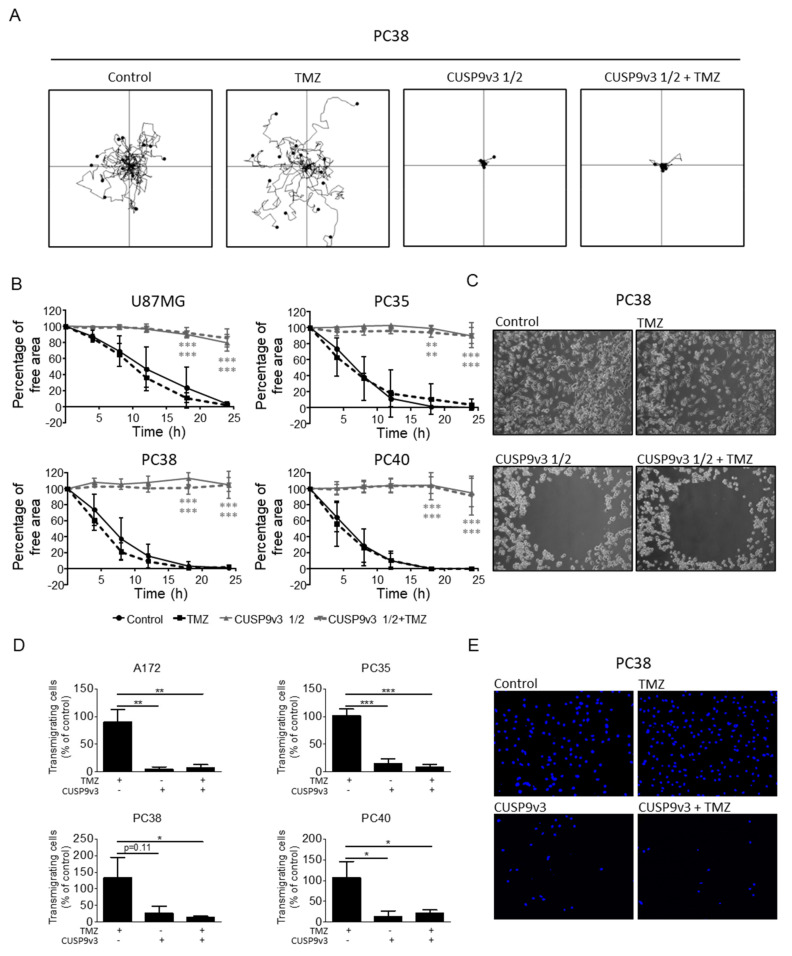
(**A**), PC38 cells were treated for 24 h as indicated. During this period, microscopic images were taken at 10× magnification every 30 min. Single-cell tracking was performed using the MtrackJ software. Wind-rose plots displaying the paths of 15 single cells per treatment condition during the 24 h observation period are shown. The tracks are zeroed to start from the same initial position. (**B**), U87MG, PC35, PC38 and PC40 cells were treated as indicated. The percentage of free area was calculated as described in Materials and Methods. Data points: mean. Bars: Standard deviation (SD). ** *p* < 0.01, *** *p* < 0.005 versus TMZ. (**C**), Representative microphotographs of PC38 cells treated for 18 h as indicated. Magnification 10×. (**D**), A172, PC35, PC38 and PC40 cells were seeded onto Transwell^®^ membranes and treated as indicated. After 8 h, representative microphotographs were taken at 10× magnification and transmigrating cells were counted (in total 9 high-power fields per treatment condition). Columns: mean. Bars: SD. * *p* < 0.05, ** *p* < 0.01, *** *p* < 0.005. (**E**), Representative microphotographs of transmigrating PC38 cells treated for 8 h as indicated prior to staining with DAPI and microscopic imaging with 10× magnification.

Enhanced migratory capacity is a major contributor to drug resistance and a hallmark feature of cancer cells. For that reason, we examined whether CUSP9v3 would affect the migratory capacity of glioblastoma cells. Due to the strong inhibitory effect of CUSP9v3 on the cellular viability of glioblastoma cells, the dosage or the treatment duration were reduced in order to not confound antimigratory and cytocidal effects. As shown in Figure 3A, treatment with CUSP9v3 reduced to one half of the original doses (CUSP9v3 1/2) led to a significant reduction of non-directed migration as assessed by time-lapse live cell imaging followed by single-cell tracking. Additional treatment with temozolomide did not enhance this effect. Notably, treatment with temozolomide alone did not result in decreased migratory capacity. We verified these observations by using a Radius^TM^ assay (Figure 3B,C). Again, treatment with CUSPv3 1/2 in the presence or absence of temozolomide exerted significant antimigratory activity while none such was perceived following single therapy with temozolomide.

We next addressed the question whether CUSP9v3 also affects directed migration. To this purpose, Transwell^®^ assays were performed (Figure 3D,E). Treatment with CUSP9v3 caused a statistically significant decrease in the number of transmigrating A172, PC35 and PC40 glioblastoma cells after 8 h. In PC38 cells, the antimigratory effect following CUSP9v3 treatment alone did not reach a statistically significant level but was further enhanced by additional treatment with temozolomide.

### 2.6. Compassionate Use Experience

Following the encouraging results from our initial in vitro studies with CUSP9v3 and due to our expectation of the benign nature of the drugs that are involved, we decided to offer the CUSP9 approach to patients with heavily pre-treated recurrent glioblastoma WHO grade IV who were ineligible for re-resection, more cytotoxic chemotherapy or clinical trial participation (the basic patient characteristics are presented in Table 2). CUSP9v3 was offered to 7 patients in this study. One patient on whom we report here had to use the antipsychotic/antidepressant drug quetiapine (25 mg daily) instead of ritonavir—resulting in a regimen termed CUSP9v3r/q—due to a pre-existing psychiatric condition.

### 2.7. Safety and Tolerability of CUSP9v3 or CUSP9v3r/q

The Common Terminology Criteria for Adverse Events (CTCAE) represent the general standard for describing drug-induced toxicity in patients. In their latest version (4.0), the CTCAE consist of 26 categories with a large variety of singular adverse events in each category. In our retrospective analysis, we decided to limit the considered adverse events to a group of 12 that was deemed particularly relevant based on an exhaustive literature review (Table 3). There were no grade 5 adverse events. The most common grade 3 or grade 4 adverse events were related to liver enzyme elevations: alanine transaminase (n = 3; 38%), aspartate aminotransferase (*n* = 1; 13%) and γ-glutamyl transferase (n = 2; 25%). The second most frequently occurring grade 3 or grade 4 adverse events were infections (n = 2; 25%) followed by hematological abnormalities (anemia, leukocytopenia or thrombocytopenia (each n = 1; 13%)).

### 2.8. CUSP9v3 Affects Contrast Enhancement on Magnetic Resonance Imaging (MRI)

**Figure 4:**
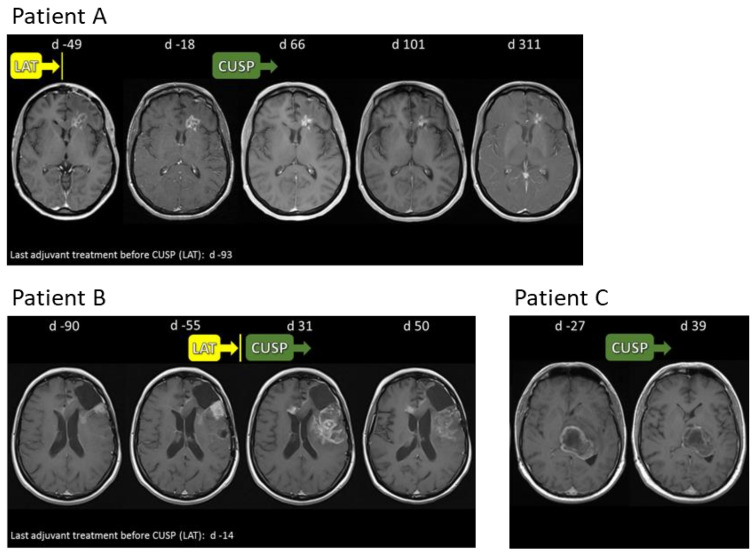
Representative T1-weighted MRIs of patients (**A**–**C**) after application of contrast media. LAT: Last adjuvant treatment before initiation of CUSP.

While patients receiving CUSP9v3 or CUSP9v3r/q on a compassionate use basis were not subjected to a strict follow-up protocol, regular MRIs were performed for clinical monitoring purposes. During the observation period, reduced contrast enhancement on T1-weighted MRI was noted in one patient receiving CUSP9v3r/q and in six of seven patients treated with CUSP9v3. For three patients, representative MRIs are shown in Figure 4. Patient A displayed a reduced contrast enhancement of the left frontal lesion by day 101 after initiation of CUSP9v3. By day 50, patient B also showed reduction of contrast enhancement. Patient C had decreased lesional contrast enhancement by day 39.

## 3. Discussion

In this work, we present our in vitro and initial clinical experiences with CUSP9v3 in glioblastoma. While others have reported the use of polypharmacological regimens including repurposed drugs in this setting, the CUSP approach is unique in that it combines far more drugs than previously used. Moreover, all of the drugs that were added to low-dose metronomic temozolomide were repurposed in a “hard” way, i.e., by using originally non-oncological drugs against an oncological condition.

Our in vitro data show that CUSP9v3 has profound antineoplastic activity across a variety of different glioblastoma models. This activity includes cytocidal, anti-proliferative and antimigratory actions. Notably, these effects were for the most part independent of additional treatment with temozolomide. Given this finding, one could question the rationale of maintaining temozolomide at low dose as part of this treatment regimen. What these experiments do not take into account is the metabolic processing temozolomide undergoes in a mammalian organism and the potential formation of more active metabolites. Therefore, we cannot exclude temozolomide-related benefits in patients receiving CUSP9v3. Based on the preliminary report of our experiences with CUSP9v3 and CUSP9v3r/q that was presented at the SNO meeting in 2017 [11], Skaga et al. examined the activity of CUSP9v3r/q in vitro [12]. In concordance with our data, treatment with CUSP9v3r/q had a significant inhibitory effect on cellular viability and sphere formation in three different glioma stem-like cell lines. When they further extended their studies to a total of 15 glioma stem-like cell cultures, Skaga et al. found a heterogenous pattern of response. The seven cell cultures that responded most sensitively were all of proneural subtype. In our study, under the conditions that we used, all cell lines showed a strong response towards treatment with CUSP9v3. While we cannot confirm the proneural subtype to be especially sensitive to CUSP9v3, this discrepancy is most likely due to the fact that we used different drug concentrations than Skaga et al. The drug levels used in our study were chosen based on literature reports on the respective drug levels achievable in patients whereas Skaga et al. used drug concentrations determined by in vitro titration assays. Notably, when we reduced the concentrations of the CUSP9v3 drugs to 1/10 of the original concentrations, A172 and T98G established glioblastoma cells as well as three glioma stem-like cells showed considerably reduced responses. As a result of this observation, we initiated studies to explore further refinements of the CUSP9 strategy in vitro. For instance, we examined a combined approach of CUSP9v3 and a targeted agent [13]. In this study, when CUSP9v3 was combined with the Bcl-2/Bcl-xL inhibitor ABT-263, synergistic inhibition of cellular viability and strong pro-apoptotic activity was noted among a broad panel of established and primary cultured glioblastoma as well as glioblastoma stem-like cells, despite a dose reduction down to 1/20th of the original CUSP9v3 dose. Similar to the results of the present study, concomitant treatment with temozolomide in a clinically relevant concentration was for the most part of no additional benefit. Furthermore, when this drug combination was extended by an inhibitor of RAC1, the migratory activity of different glioblastoma cells was even more strongly reduced. As it is evident from the history of the CUSP9 strategy, its development is a dynamic process which entails cautious rational and well-balanced drug substitutions and/or additions.

An important question that remains to be addressed is whether all 10 drugs are equally necessary to attain maximal CUSP9 activity. Beyond extensive in vitro testing of all possible drug combinations, much more complex issues need to be studied, e.g., drug effects on the tumor microenvironment and the role of drug metabolites that only occur in vivo.

Our study of CUSP9v3/v3r/q follows the same principal lines as the CLOVA regimen that was applied to seven patients with recurrent glioblastoma and a low Karnofsky Performance Score and involved inhibiting glycogen synthase kinase 3-β using a drug combination of standard temozolomide (200 mg/m^2^ body surface area/d for 5 d every 4 weeks) with adjunct cimetidine (800 mg/d), lithium carbonate (400 mg/d), olanzapine (10 mg/d) and valproic acid (800 mg/d)—all generic and inexpensive drugs that have shown preclinical evidence of anti-glioblastoma action [14]. With cimetidine being marketed to lower gastric acid production, lithium to treat psychiatric conditions, olanzapine to treat psychosis and valproate to treat seizures, all possess ancillary attributes to diminish glycogen synthase kinase 3-β activity. In vitro, proliferation of established glioblastoma cells was only slightly reduced by the CLOVA combination alone, but more pronounced effects were noted in combination with temozolomide. Moreover, cellular invasion was significantly impaired following treatment with the CLOVA combination [14]. In a murine orthotopic glioblastoma model, treatment with CLOVA led to a significant survival advantage. In the subsequent phase I/II clinical trial 1 patient achieved partial response and 6 patients had stable disease. The medication was well-tolerated, and median overall survival after tumor recurrence was 11.2 months compared to 4.3 months in matched historical controls receiving only temozolomide maintenance therapy [14].

The COMBAT trial is another example of a multi-drug approach including ancillary drugs [15]. In this trial, 74 children with advanced solid malignancies were treated with temozolomide and etoposide along with celecoxib, vitamin D, fenofibrate and retinoic acid. Thirteen of these patients had a medulloblastoma, i.e., an embryonal malignant brain tumor. Aside from hematological toxicity, this treatment regimen was generally well-tolerated. Median overall survival for all patients in this study was 15.4 months while in the subgroup of patients with medulloblastoma, median overall survival reached 40.8 months.

While we have not tested the effects of CUSP9v3 on non-neoplastic cells such as astrocytes or neurons in vitro, we did study the safety of this regimen in 8 patients with recurrent glioblastoma in a last-ditch effort. With respect to tolerability of CUSP9v3 in our small series, the results are comparable to the findings in larger clinical trials evaluating other glioblastoma treatments. More detailed information on CUSP9v3-related AEs was recently reported from the CUSP9v3 phase Ib/IIa clinical trial. In line with our initial experience, a favorable safety profile was found [16].

Robust in vitro anti-glioblastoma activity, a sound rationale presented here and in the two background papers, the low side effect burden and evidence of CUSP9v3-induced radiological changes in our small compassionate use patient series all supported the development of a phase Ib/IIa CUSP9v3 trial (NCT02770378). A multi-center, randomized controlled phase II clinical trial of CUSP9v3 in newly-diagnosed glioblastoma is currently in preparation.

## 4. Materials and Methods

### 4.1. Reagents

Aprepitant (Selleckchem, Houston, TX, USA.; stock: 100 mg/mL, final conc.: 1 µg/mL), auranofin (Sigma Aldrich, St. Louis, MO, USA; stock: 5 mg/mL, final conc.: 0.9 µg/mL), celecoxib (Sigma Aldrich; stock: 20 mg/mL, final conc.: 1.8 µg/mL), disulfiram (Sigma Aldrich; stock: 5 mg/mL, final conc.: 0.05 µg/mL), itraconazole (Abcam, Cambridge, U.K.; stock: 20 mg/mL, final conc.: 0.5 µg/mL), ritonavir (Sigma Aldrich; stock: 10 mg/mL, final conc.: 12.3 µg/mL) and temozolomide (Sigma Aldrich; stock: 20 mg/mL, final conc.: 6 µg/mL) were dissolved in dimethyl sulfoxide (DMSO). Captopril (Sigma Aldrich; stock: 50 mg/mL, final conc.: 1.3 µg/mL) and sertraline (Sigma Aldrich; stock: 3.8 mg/mL, final conc.: 0.03 µg/mL) were dissolved in H_2_O. All stock solutions were stored at −20 °C. For all experiments, final concentrations of DMSO were below 0.1% (*v*/*v*).

### 4.2. Cell Cultures and Growth Conditions

U87MG, A172 and T98G human glioblastoma cell lines were obtained from the American Type Culture Collection (Manassas, VA, USA) and were cultured in Dulbecco’s modified Eagle’s medium (DMEM; GIBCO, Invitrogen, Paisley, UK) containing 10% fetal bovine serum (FBS), 100 IU/mL penicillin, 100 µg/mL streptomycin, 4 mM glutamine, 1 mM sodium pyruvate (GIBCO, Invitrogen, Grand Island, NY, USA) as described before [17]. The initial stocks had been expanded, frozen and stored in liquid nitrogen. Fresh aliquots were thawed every 6 weeks. Primary cultured glioblastoma cells (SC35, SC38 and SC40) had been generated from tumor resections performed at our institution as previously described [18,19]. The glioma stem-like phenotype of SC35, SC38 and SC40 cells was preserved by maintaining cells as sphere cultures in DMEM/F-12 (HAM) medium (Gibco, Life Technologies, Darmstadt, Germany) supplemented with human recombinant epidermal growth factor (Biomol GmbH, Hamburg, Germany), human recombinant basic fibroblast growth factor (Miltenyi Biotec GmbH, Bergisch Gladbach, Germany) and serum-free neuron culture supplement B27 (Gibco, Life Technologies). Differentiated cells (PC35, PC38 and PC40) were derived from the respective glioma stem-like cell spheres by allowing adherence and growth in DMEM in the presence of 10% FBS and were maintained for a maximal duration of 10 weeks. Patient’s or next of kin’s consent was obtained, and research procedures were approved by the institutional review board (approval no. 162/10). All cell lines were incubated at 37 °C in a water-saturated atmosphere containing 5% CO_2_.

### 4.3. Cell Viability Assays

In order to examine cellular proliferation, 3-[4, 5-dimethylthiazol-2-yl]-2, 5-diphenyltetrazolium bromide (MTT) assays and cell count analyses were performed [20,21]. For MTT assays, 1.5 × 10^3^ cells/well were seeded in 96-well flat-bottomed plates and allowed to attach overnight at 37 °C prior to changing the medium to DMEM supplemented with 1.5% FBS and treatment with compounds or the corresponding solvents. At defined time points, the medium was aspirated and 100 µL MTT solution were added to the wells prior to incubation at 37 °C for 3 h. The reaction was stopped by adding 100 µL of 100% isopropanol (Sigma-Aldrich), and optical densities (OD) were measured at 550 nm using an automated microplate reader. Percent viability was expressed as (OD_compound_-OD_blank_)/(OD_solvent_-OD_blank_). For cell count determinations, 1 × 10^4^ cells/well were seeded in 12-well plates. Similar to the MTT assays, cells were allowed to attach, and medium was changed prior to adding the different compounds. At distinct time points, the medium was aspirated, the cells were enzymatically detached (Trypsin/EDTA, Biochrom AG, Berlin, Germany) and cell numbers were determined with a cell counter.

### 4.4. Soft Agar Assay

Anchorage-independent growth was assessed as previously described [21,22]. Briefly, 6-well plates were coated with a base layer of 0.9% low-melting agarose (Biozym Scientific GmbH, Hess. Oldendorf, Germany) containing 10% FBS, antibiotics and the respective reagents or corresponding solvents. A layer of 0.35% agarose containing the same supplements as the base layer plus 2 × 10^4^ cells/well was placed on top of the base layer prior to incubation for 21 d at standard culture conditions. Microscopic images were taken at 4-fold magnification. The largest diameter of the colonies was measured, and colonies with a diameter exceeding 150 µm (T98G, SC35, SC38 and SC40) or 200 µm (U87MG, PC35, PC38 and PC40) were counted.

### 4.5. Spheroid Assay

Spheroids were established to assess the effects of CUSP9v3 in a 3-dimensional growth setting. In 96-well plates, 0.35 × 10^5^ cells/well were resuspended in 20 µL of a mixture of 80% Matrigel and 20% DMEM prior to incubation for 1 h at 37 °C. Afterwards, the cell/Matrigel matrix was gently transferred to 12-well plates containing DMEM (1.5% FBS) alone or DMEM (1.5% FBS) with the respective treatments depending on the corresponding model. Two therapeutic models were examined. In the chemoprevention model, spheroids were treated immediately after transfer of the cell/Matrigel matrix followed by treatments on d7, d9 and d12. In the established tumor model, spheroids were allowed to grow for 7 days before starting treatments. Spheroids were treated on d7, d9 and d12. For quantification, CellTiter-Glo^®^ assays were performed. To this purpose, spheroids suspended in 100 µL of medium were transferred to opaque-walled 96-well plates prior to adding 100 µL of the CellTiter-Glo^®^ solution followed by incubation for 10 min at RT and measurement of luminescence.

### 4.6. Cell Migration Assays

Cell migration was examined by a transmembraneous migration (Transwell^®^) assay and a Radius^TM^ assay. Cell motility was evaluated by time-lapse live cell microscopy imaging [21,22].

The Radius^TM^ cell migration assay was purchased from Cell Biolabs, Inc. (San Diego, CA, USA) and performed according to the manufacturer’s instructions. Briefly, 24-well plates were treated with gel pretreatment solution for 20 min and washed once prior to seeding 8 × 10^4^ cells/well in DMEM containing 10% FBS. After 24 h, the supernatant was aspirated and the gel removal solution was added prior to incubation at 37 °C for 30 min. The wells were washed twice with DMEM containing 1.5% FBS. Microscopic images were taken at 10-fold magnification at various time points. The percentage of free area was determined using the NIH ImageJ software (http://imagej.nih.gov/ij, accessed on 10 August 2020) and was calculated as (free area at 0 h/free area time x) ×100.

For the Transwell^®^ assay, 3 × 10^4^ cells were seeded in DMEM containing 1.5% FBS onto membranes (Corning Incorporated, Corning, NY, USA) with a pore size of 8 µm, and intrinsically migrated towards medium containing 10% FBS. Experiments were carried out according to the manufacturer’s recommendations. After 8 h, the upper side of the membrane was wiped and washed three times with phosphate-buffered saline (PBS). The cells on the bottom side of the membrane were then fixed with methanol and stained with 4′6-diamidino-2-phenylindole (DAPI) prior to mounting. The number of migrated cells was determined by counting one high-power field at 10-fold magnification in triplicate for each treatment condition.

For time-lapse live cell microscopy imaging, 4 × 10^4^ cells/well were seeded onto 12-well plates, and microscopic images at 10-fold magnification were taken with a live imaging inverted video microscope (IX81, Olympus, Hamburg, Germany) every 30 min for a total observation time of 24 h. During this period, cells were kept at standard culture conditions (37 °C, 5% CO_2_, water-saturated atmosphere). Single-cell tracking was performed with the MtrackJ plugin (www.imagescience.org/meijering/software/mtrackj/, accessed on 10 August 2020) for the NIH ImageJ software. Normalized “wind-rose” plots were generated with the chemotaxis and migration tool from Integrated BioDiagnostics (Martinsried, Germany, www.ibidi.com, accessed on 10 August 2020).

### 4.7. Western Blot Analysis

Specific protein expression in cell lines was determined by Western blot analysis using anti-human caspase-3 (1:1000; #9662, Cell Signaling Technology (CST), Danvers, MA, USA) and anti-β-actin (1:2000, clone AC15; Sigma Aldrich) as described before [15,16]. Secondary antibodies were purchased from CST (#7076S, #7074S). Briefly, for cell lysis a buffer was used containing 150 mM NaCl, 1% Triton X-100, 10% glycerol, 30 mM Tris–HCl (pH 7.4), 200 mM phenylmethylsulfonyl fluoride, 2 mM dithiothreitol, 1 mM 3-glycerophosphate, 1 mM Na_2_VO_3,_ 50 mM NaF and the Complete Protease Inhibitor Cocktail (Roche Diagnostics GmbH, Mannheim, Germany). The cell lysates were transferred to Eppendorf tubes and denaturation was performed at 70 °C for 10 min prior to storage at −20 °C. Fifty µg per sample were separated on SDS-PAGE and transferred to a nitrocellulose membrane (Amersham, Chicago, IL, USA) before incubation with specific antibodies. The Pierce^TM^ enhanced chemiluminescence Western blotting substrate was used for detection of target proteins.

### 4.8. Statistical Analysis

Statistical significance was assessed by one-way ANOVA followed by Newman-Keuls post hoc analysis using PRISM (GraphPad Software, La Jolla, CA, USA) version 5.04. A *p* ≤ 0.05 was considered statistically significant.

## Figures and Tables

**Table 1:** Evolution of the different CUSP9 regimes.

CUSP9	CUSP9*	CUSP9v3	CUSP9v3r/q ^1^
aprepitant -antiemetic	aprepitant	aprepitant 80 mg; 1×/d	aprepitant
artesunate -antimalarial	artesunate	minocycline -antibiotic 100 mg; 2×/d	minocycline
auranofin -antirheumatic	auranofin	auranofin 3 mg; 2×/d	auranofin
captopril -antihypertensive	captopril	captopril 50 mg; 2×/d	captopril
disulfiram- alcohol deterrent	disulfiram	disulfiram 250 mg; 2×/d	disulfiram
copper gluconate -food supplement ^2^	celecoxib -NSAID ^3^	celecoxib 400 mg; 2×/d	celecoxib
ketoconazole -antifungal	itraconazole -antifungal	itraconazole 200 mg; 2×/d	itraconazole
nelfinavir -antiretroviral	ritonavir -antiretroviral	ritonavir 400 mg; 2×/d	quetiapine -atypical antipsychotic 25 mg; 1×/d
sertraline -antidepressant	sertraline	sertraline 100 mg; 2×/d	sertraline
temozolomide -chemotherapeutic	temozolomide	temozolomide 20 mg/m^2^; 2×/d	temozolomide

^1^ Previously referred to as CUSP9v4, ^2^ potential disulfiram enhancer, ^3^ non-steroidal anti-inflammatory drug.

**Table 2:** Individual patient characteristics.

	Age (Years)	Gender	MGMT-Status	IDH-Status
Patient 1	69	M	Pos.	WT
Patient 2	56	M	Neg.	WT
Patient 3	42	F	Neg.	Mut
Patient 4	51	M	Neg.	WT
Patient 5	41	F	Pos.	WT
Patient 6	43	M	Neg.	WT
Patient 7	40	M	Neg.	WT
Patient 8	44	M	Neg.	WT

**Table 3:** Grade 3 or grade 4 adverse events.

Adverse Event	N (%)
ALT increased	3 (38)
γGT increased	2 (25)
AST increased	1 (13)
Infection	2 (25)
Anemia	1 (13)
Leukocytopenia	1 (13)
Thrombocytopenia	1 (13)
PTT prolongation	0 (0.0)
Creatinine increase	0 (0.0)
Nausea	0 (0.0)
Vomiting	0 (0.0)
Thromboembolus	0 (0.0)

ALT: alanine aminotransferase, AST: aspartate aminotransferase, γGT: gamma-glutamyl transferase, PTT: partial thromboplastin time.

## Data Availability

The data supporting the results reported in the article are deposited at the Translational Brain Tumor Research Laboratory, Department of Neurological Surgery, Ulm University Medical Center, Albert-Einstein-Allee 23, D-89081 Ulm, Germany.

## Supplementary Materials

The following are available online at https://www.mdpi.com/article/10.3390/ph14121241/s1, Figure S1: Effects of CUSP9v3 1/10 on the cellular viability of glioblastoma cells.
