# Supplementary material for: In Vitro and Clinical Compassionate Use Experiences with the Drug-Repurposing Approach CUSP9v3 in Glioblastoma
Source: Pharmaceuticals (Basel). 2021 Nov 29;14(12):1241. doi: 10.3390/ph14121241 (PMC8708851; doi:10.3390/ph14121241)
Supplement: Supplementary file 1 [file pharmaceuticals-14-01241-s001.zip › pharmaceuticals-1262199-supplementary.pdf]

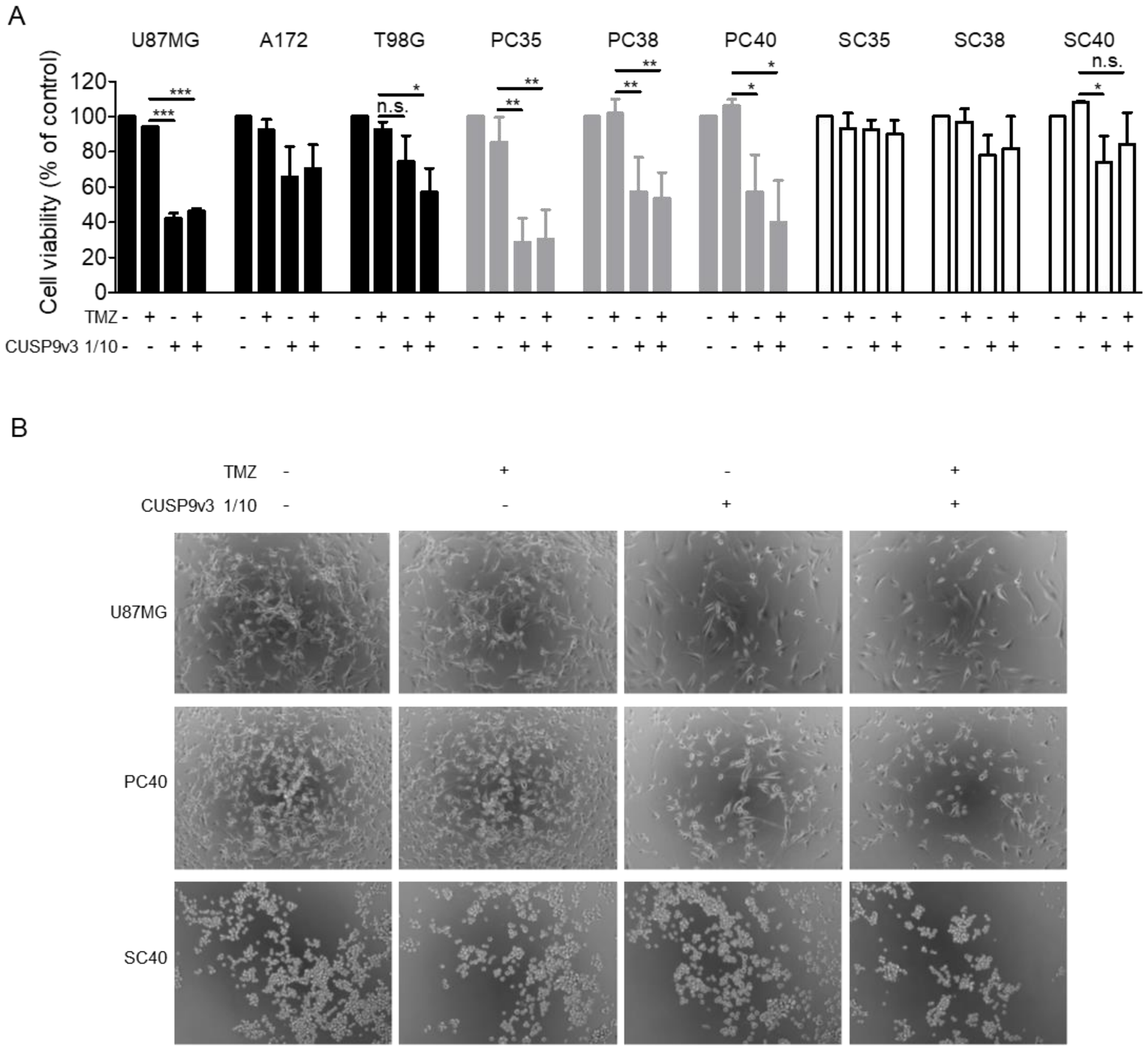

Figure S1. A, U87MG, A172, T98G established glioblastoma cell lines, PC35, PC38 and PC40 glioblastoma primary cultures and SC35, SC38 and SC40 glioblastoma stem-like cells were treated with solvent, temozolomide (TMZ), CUSP9v3 1/10 or CUSP9v3 1/10 plus TMZ for 144 h. Cellular viability was determined by MTT assay. Data represent three independent experiments. Columns: mean. Bars: standard deviation. * $p<0.05$, ** $p<0.01$, *** $p<0.005$. B, Representative microphotographs of U87MG, PC40 and SC40 glioblastoma cells treated for 144 h as indicated. Magnification 20 x.
